# Diabetic Macular Edema Treatment with Bevacizumab Does Not Depend on the Retinal Nonperfusion Presence

**DOI:** 10.1155/2021/6620122

**Published:** 2021-02-26

**Authors:** Bogumiła Sędziak-Marcinek, Sławomir Teper, Elżbieta Chełmecka, Adam Wylęgała, Mateusz Marcinek, Mateusz Bas, Edward Wylęgała

**Affiliations:** ^1^Chair and Department of Ophthalmology, Faculty of Medical Sciences in Zabrze, Medical University of Silesia, 40-760 Katowice, Poland; ^2^Department of Statistics, Department of Instrumental Analysis, Faculty of Pharmaceutical Sciences in Sosnowiec, 41-200 Sosnowiec, Medical University of Silesia, Katowice, Poland; ^3^Health Promotion and Obesity Management Unit, Department of Pathophysiology, Faculty of Medical Sciences in Katowice, Medical University of Silesia, 40-728 Katowice, Poland; ^4^Department of Urology, Faculty of Medical Sciences in Katowice, Medical University of Silesia, 40-760 Katowice, Poland; ^5^Faculty of Biomedical Engineering, Silesian University of Technology, 41-800 Zabrze, Poland

## Abstract

This study evaluated the relationship between the retinal nonperfusion area (NPA) presence and the effectiveness of bevacizumab treatment (IVB) in patients with diabetic macular edema (DME). It also tested the prognostic usefulness of ultra-wide-field fluorescein angiography (UWFFA) and OptosAdvance software for diabetic retinopathy monitoring. Eighty-nine patients with DME with a macular central subfield thickness (CST) ≥ 250 *μ*m, with (*N* = 49 eyes) and without (*N* = 49 eyes) retinal NPA, underwent nine bevacizumab injections over 12 months. NPA distribution, leakage area distribution, microaneurysm (MA) count, macular CST, diabetic retinopathy severity, and best-corrected visual acuity (BCVA) were assessed. The results show that bevacizumab reduced the macular CST from 420 to 280 *μ*m (*p* < 0.001) and improved BCVA (*p* < 0.001) by about 10 ETDRS letters in both groups of patients. Additionally, the therapy reduced total retinal NPA from 29 (14-36) mm^2^ to 12 (4-18) mm^2^ (Me (Q1-Q3); *p* < 0.001) in patients with diagnosed nonperfusion. The effect of the therapy measured with vascular leakage, MA count, BCVA_relative_, and CST_relative_ strongly depended on the zone of the retina and the NPA distribution. We conclude that the bevacizumab treatment had a positive effect on DME and BCVA in both study groups and on the size of retinal NPA in patients with retinal nonperfusion.

## 1. Introduction

Diabetic retinopathy (DR) is considered the most common microvascular complication of diabetes [1]. Some authors suggested that almost all T1DM (type 1 diabetes mellitus) patients would have some degree of retinopathy 20 years from T1DM diagnosis, as would more than 80% of insulin-treated T2DM (type 2 diabetes mellitus) patients and 50% of those not requiring insulin [2, 3]. The diabetic retinopathy lesions may occur in different areas of the retina influencing the severity and progression of this disease [4, 5]. It was shown that the extent of capillary nonperfusion can increase from the peripapillary retina in ascending order and that the areas of nonperfusion occur in the midperiphery [4]. DR lesions first develop in the peripheral retina, and they are predominantly associated with a significantly larger area of retinal nonperfusion [4, 5]. Moreover, Silva et al. reported that the extent of capillary nonperfusion in the midperiphery increased as DR progressed [4].

The most common cause of vision loss in diabetic patients is diabetic macular edema (DME). DME is defined as a retinal thickening at the center of the macula or the thickening approaching this center [6]. The pathophysiological process is related to a decrease in the retinal oxygen saturation, which makes retinal capillaries hyperpermeable [7]. The whole process is mediated by the upregulation of vascular endothelial growth factor (VEGF) and subsequent deterioration of retinal capillary autoregulation [7]. DME-related nonperfusion increases the risk of more complex ophthalmic disease development. Impaired macular perfusion is frequently asymptomatic until the later stages, leading to more acute and severe vision loss. Thus, it is essential to recognize and quantify these microvascular transformations to assess disease severity and implement optimal treatment protocol to prevent vision loss [8].

Bevacizumab (IVB) is a monoclonal, humanized, full-length antibody inhibiting vascular endothelial growth factor (VEGF). Currently, bevacizumab is widely used in DME treatment as an off-label drug [9–11].

Ultra-wide-field fluorescein angiography (UWFFA) allows producing high-resolution images with a 200° field view covering >80% of the retinal surface while traditional angiograms cover only 30-50° field view of the retina. The newest UWFFA stereographical images allow for correction of the inherent peripheral retinal distortion. This allows for accurate measurements of both posterior and peripheral areas of the retina [12–14]. UWFFA imaging has become the standard technique of peripheral nonperfusion assessment in retinal vascular diseases and is used to study the relationship between retinal nonperfusion and DME severity [4, 15].

In this study, we evaluated the relation between the retinal nonperfusion area presence and the effectiveness of bevacizumab treatment in patients with diabetic macular edema that were not subjected to the previous treatment. We also evaluated the usefulness of UWFFA imaging for prognostic purposes in patients with diabetic macular edema.

## 2. Materials and Methods

### 2.1. Permissions and Ethical Statements

The study was approved by the Ethics Committee of the Medical University of Silesia (decision KNW/0022/KB1/125/I/18/19). The study was conducted following the Declaration of Helsinki. The participants were informed about the study purpose, the study protocol, and the benefits and possible risks related to the study. The written consent had been obtained from all participants enrolled in the study.

### 2.2. Study Group

The participants were recruited from the ophthalmological outpatient clinic of the Clinical Department of Ophthalmology, Faculty of Medical Science, Medical University of Silesia. The inclusion and exclusion criteria in the study are presented in Table 1.

The patients' recruitment, diagnostics, and intravitreal treatment were carried out in the outpatient clinic throughout 2018-2020.

Based on studies by Ekinci et al. that reported CST changes after bevacizumab treatment [16], the necessary sample size for this study was calculated as 25 samples. Since the purpose of this study was to differentiate between the two groups of subjects, with and without nonperfusion, a group of 98 patients was selected for the study: 49 with nonperfusion and 49 without nonperfusion. With this sample size, it was possible to meet the assumption of a minimum sample size for a significance level of 0.05 and a test power of 0.8.

### 2.3. Recruitment and Diagnostics of the Participants

Patients of the ophthalmological outpatient clinic were interviewed and initially examined during the routine appointment.

The interview consisted of a chart survey, and the following data were recorded: sex, age, type and duration of diabetes, serum glycated hemoglobin (HbA1c) level, and the following qualitative variables such as hypertension, current medication with blood thinners, and renal failure.

The initial examination was done with a slit lamp and was aimed at assessing the anterior and posterior segments of the eye.

#### 2.3.1. Retinal Nonperfusion and Diabetic Macular Edema Diagnostics

The patients were subjected to further diagnostics to identify those with retinal nonperfusion and diabetic macular edema.

Ultra-wide-field fluorescein angiography (UWFFA) allowed analyzing the distribution of nonperfusion areas (NPA), distribution of leakage area, and microaneurysm (MA) count and assessing the severity of the diabetic retinopathy.

Swept-source optical coherence tomography (SS-OCT) enabled the assessment of diabetic macular edema by measuring central subfield thickness (CST).

The patients with DME and with central subfield thickness (CST) ≥ 250 *μ*m were enrolled in the study and divided into two subgroups according to the retinal nonperfusion presence: patients with retinal nonperfusion (*N* = 49 eyes) and patients without retinal nonperfusion (*N* = 49 eyes). UWFFA and SS-OCT imaging allowed assessing the effectiveness of the bevacizumab treatment, monitoring the changes in retinal nonperfusion, and assessing the usefulness of UWFFA for retinal nonperfusion prognosis.

### 2.4. Bevacizumab Treatment Protocol

Ninety-eight eyes were qualified for bevacizumab treatment. The intravitreal injections were performed in the ophthalmological outpatient clinic of the Clinical Department of Ophthalmology of Faculty of Medical Science of the Medical University of Silesia. The injections were administered by an ophthalmologist. The eyes were locally anesthetized with proxymetacaine hydrochloride, disinfected with 5% iodine povidone, and then injected with 0.5 mg/0.05 ml bevacizumab (Avastin, Roche, Swiss). The first five injections were administered every month, and the next four injections were administered every two months. Each eye received 9 injections over 12 months to reach the loading dose of bevacizumab [11, 17, 18].

Before the first and one month after the last injection (on the 13th month of the study), the patients were subjected to the UWFFA and BCVA test. Also, before each injection, the central subfield thickness (CST) was measured to assess diabetic macular edema.

### 2.5. Diagnostic Methodology

#### 2.5.1. Ultra-Wide-Field Fluorescein Angiography (UWFFA)

**Figure 1:**
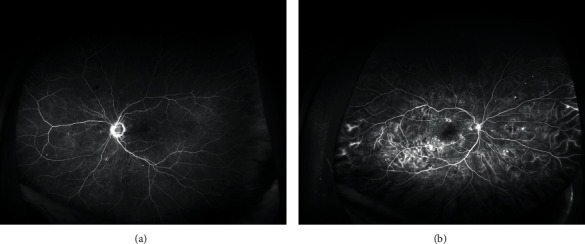
The ultra-wide-field fluorescein angiography (UWFFA) images of diabetic macular edema (DME) (a) without retinal nonperfusion and (b) with retinal nonperfusion. The images were obtained using the Optos California P200DTx scanning laser ophthalmoscope and the OptosAdvance Software v4.2.31.

Ultra-wide-field fluorescein angiography (UWFFA) images were obtained using the Optos California P200DTx (Optos, Dunfermline, Scotland, UK) scanning laser ophthalmoscope imaging system equipped with the OptosAdvance Software v4.2.31 (Figures 1(a) and 1(b)). The preparation and image analysis were done according to the methodology by Fan et al. and Fang et al. [19, 20].

*(1) Preparation*. The pupils were dilated by topical administration of tropicamide 1% (Polpharma, Starogard Gdański, Poland) and phenylephrine 2.5% to the conjunctival sac. Sodium fluorescein (250 mg; SERB, Paris, France) was administered intravenously *via* the median ulnar vein, and the images were captured at 45 s, 2 min, and 5 min of fluorescein angiography (early, middle, and late phase, respectively).

*(2) Image Analysis*. The images obtained for each eye were reviewed to check the image quality and its eligibility for the quantitative variables and diabetic retinopathy severity assessment.

The initial and after-treatment UWFFA images were analyzed by two trained UWFFA graders/ophthalmologists using OptosAdvance software v4.2.31 that allows for accurate measurements of the total visible retinal area in square millimeters (mm^2^) and adjusts for peripheral distortion. The graders were allowed to adjust the contrast and brightness to obtain optimal visualization. They analyzed each image and each step until a unanimous decision was reached. On the obtained UWFFA images, the graders manually delineated the peripheral extent of the visible retina—the total visible retina. The total visible retina was defined as the region where the blood vessels of the retina were visible and easily assessed for the capillary nonperfusion presence. The peripheral artifacts (i.e., eyelids and eyelashes) and the areas of poor image quality were excluded from this region. Pairs of UWFFA images (initial and after treatment) were applied on top of each other so their surfaces were identical and to make sure that no errors in before- and after-treatment image analyses were possible. After the delineation of the total visible retina, the graders manually delineated the border of the nonperfusion area (NPA) and the leakage area.

**Figure 2:**
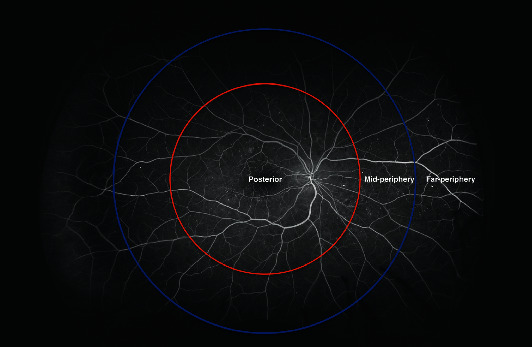
Zones of the retina relative to the center of the fovea: posterior zone (radius < 10 mm), midperiphery zone (radius = 10 − 15 mm), and far-periphery zone (radius > 15 mm). The ultra-wide-field fluorescein angiography (UWFFA) image was obtained using the Optos California P200DTx scanning laser ophthalmoscope and the OptosAdvance Software v4.2.31.

**Figure 3:**
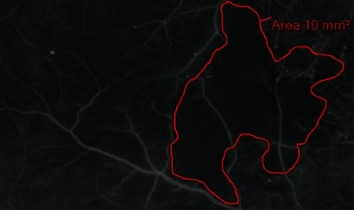
Nonperfusion area delineation and measurement on the ultra-wide-field fluorescein angiographic (UWFFA) image. The nonperfusion area is outlined in red. The size of the nonperfusion area (mm^2^) was measured automatically using the OptosAdvance software v4.2.31.

*(3) Nonperfusion Area (NPA) Distribution*. The nonperfusion area was assessed using early phase UWFFA images (captured at 45 s of fluorescein angiography). First, the prespecified custom grid was applied to the images. The grid consisted of two circles of 10 and 15 mm radii, centered on the fovea. The circles divided the UWFFA image into three zones: a posterior (<10 mm radius), a midperiphery (10-15 mm radii), and a far periphery (>15 mm radius) (Figure 2). Then, the graders manually delineated the nonperfusion areas in the posterior, midperiphery, and far-periphery zones (Figure 3). The foveal area was excluded from the posterior zone analysis due to the possibility of bias. The nonperfusion area (NPA) was identified by the absence of retinal arterioles and/or capillaries with overall hypofluorescence relative to the overall background. Additionally, the image was compared to the respective fundus color image in search of intraretinal hemorrhages that could suppress fluorescence and simulate nonperfusion areas. Finally, the surface of nonperfusion areas in each zone was counted against the grid and expressed in square millimeters (mm^2^).

**Figure 4:**
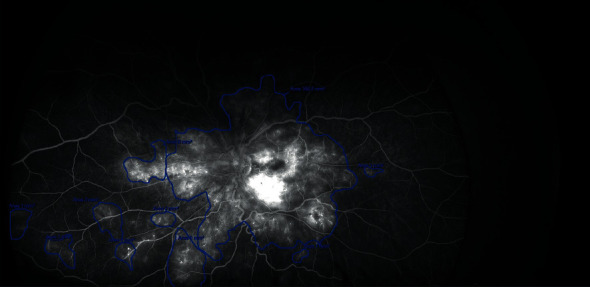
Leakage area delineation and measurement on the ultra-wide-field fluorescein angiographic (UWFFA) image. The leakage areas are outlined in blue. The size of leakage areas (mm^2^) was measured automatically using the OptosAdvance software v4.2.31.

*(4) Leakage Area Distribution*. The leakage area distribution was assessed using the same prespecified grid and methodology as for the nonperfusion area distribution, but the leakage area was assessed using late phase UWFFA images (captured at 5 min of fluorescein angiography) and was identified by hyperfluorescence relative to the overall background (Figure 4). Basing on the leakage area distribution, we were able to distinguish between focal and diffuse DME. Focal DME is characterized by localized leakage from microaneurysms (MA), while in diffuse DME, the leakage involves the entire circumference of the fovea and the area has no clear edges [21].

**Figure 5:**
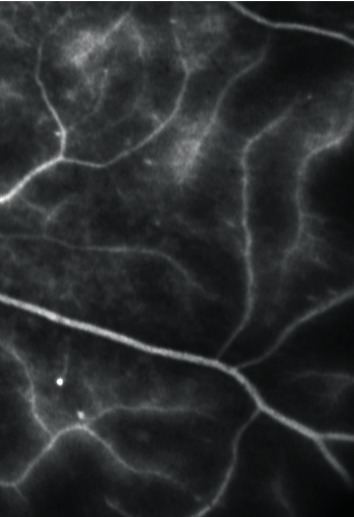
Vascular leakage on the ultra-wide-field fluorescein angiographic (UWFFA) image.

*(5) Vascular Leakage Determination*. The vascular leakage was assessed using the late phase UWFFA images (captured at 5 min of fluorescein angiography) and the same prespecified grid as the one used for the NPA and leakage area distribution. Vascular leakage was identified by late venous or arterial hyperfluorescence relative to the overall background (Figure 5).

**Figure 6:**
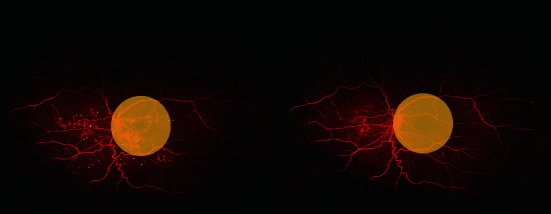
Microaneurysms (white dots) before bevacizumab therapy (left side) and after bevacizumab therapy (right side) counted by the automated platform. Yellow circles indicate the posterior retinal area. The ultra-wide-field fluorescein angiography (UWFFA) images were obtained using the Optos California P200DTx scanning laser ophthalmoscope and the OptosAdvance Software v4.2.31.

*(6) Microaneurysm (MA) Count and Microaneurysm Segmentation*. The microaneurysm (MA) count was assessed using early phase UWFFA images (captured at 45 s of fluorescein angiography) (Figure 6). The MA count segmentation was performed with a fully automated algorithm platform developed for the study by Silesian University of Technology.

First, the input UWFFA image pairs were normalized for pixel intensity values, and the histograms were equalized. Then, masks of retinal blood vessels were generated using Hessian matrices and enhanced using mathematical morphology operations (closing and dilation). Structures that were too small to be classified as vessels were removed. Next, masks of microaneurysms were generated using Hessian matrices. Masks of the retinal area were generated using previously prepared masks of retinal blood vessels. This way, the mask of the retinal area covered most of the retinal blood vessel range. The masks of microaneurysms with areas too large to be classified as microaneurysms were removed using mathematical morphology operations. Masks of microaneurysms that occurred on the retinal blood vessels or outside the retinal area were removed using previously obtained retinal blood vessel and retinal area masks. Finally, the microaneurysms occurring in the posterior and peripheral parts of the retina were counted using the MATLAB® program (The MathWorks Inc., Natick, Massachusetts, USA).

*(7) Diabetic Retinopathy Severity*. The severity of diabetic retinopathy was assessed according to the diabetic retinopathy severity scale (DRSS) as mild, moderate, or severe nonperfusion diabetic retinopathy (NPDR) based on ultra-wide photograph of the fundus of the eye [22].

#### 2.5.2. Swept-Source Optical Coherence Tomography (SS-OCT)

Swept-source optical coherence tomography images were obtained using the DRI OCT Triton tomograph (Topcon, Japan).

The images were taken using a 6 × 6 mm scanning protocol of a central macular field with an ETDRS grid to obtain thickness data. The central subfield thickness (CST; in *μ*m) was defined as the retinal thickness at the fovea. The diabetic macular edema was diagnosed when CST was ≥250 *μ*m.

### 2.6. Analysis of the Effectiveness of Bevacizumab Treatment in Particular Areas of the Retina in Patients with Retinal Nonperfusion

To assess the effects of the therapy in each zone of the retina, the relative macular central subfield thickness (CST_relative_) (equation (1)) and relative best-corrected visual acuity (BCVA_relative_) (equation (2)) were calculated for patients with diagnosed retinal nonperfusion. For the remaining variables, an analogous, general formula of equation (3) was used:

(1) $$\begin{array}{c} {\text{CST}}_{\text{relative}}=\frac{{\text{CST}}_{\text{after therapy}}-{\text{CST}}_{\text{before therapy}}}{{\text{CST}}_{\text{before therapy}}}∙100\%, \end{array}$$

(2) $$\begin{array}{c} {\text{BCVA}}_{\text{relative}}=\frac{{\text{BCVA}}_{\text{after therapy}}-{\text{BCVA}}_{\text{before therapy}}}{{\text{BCVA}}_{\text{before therapy}}}∙100\%, \end{array}$$

(3) $$\begin{array}{c} {\text{Variable}}_{\text{relative}}=\frac{{\text{variable}}_{\text{after therapy}}-{\text{variable}}_{\text{before therapy}}}{{\text{variable}}_{\text{before therapy}}}∙100\%. \end{array}$$

### 2.7. Statistical Analysis

Qualitative variables are presented using the percentages. The *χ*^2^ test was used to determine the relationship between nonmeasurable features, and then the odds ratio (OR), with 95% confidence intervals (CI), was calculated in case of a statistically significant relationship.

Quantitative variables are presented as mean and standard deviation (for data with normal distribution) or as median and lower-upper quartiles (for data with nonnormal or skewed distribution). Data distribution normality for quantitative characteristics at the beginning and at the end of the therapy was checked with the Shapiro-Wilk test and quantile plots and then the Student *t*-test for dependent samples or the nonparametric Wilcoxon pairwise test was used accordingly. In the central subfield thickness (CST) analysis, depending on the injection sequence number, an analysis of variance for repeated measurements with contrast analysis was used. The homogeneity of variances was checked with Levene's test. The variables with nonnormal distribution were analyzed using Friedman's ANOVA test. Whenever necessary, the normality of variables was improved using a logarithmic transformation. To determine the correlation between quantitative variables, Spearman's rank correlation coefficient was calculated. All statistical calculations were done using the Statistica v. 13.1 program (TIBCO, Palo Alto, USA), and statistical significance was set at *p* < 0.05.

## 3. Results

### 3.1. Retinal Nonperfusion Occurrence and Cooccurrence with Diabetic Retinopathy Parameters

Our analyses (please see Table 2 for reference) showed that the occurrence of retinal nonperfusion was independent of gender (*p* = 0.838) and the treated eye (*p* = 0.418). However, it correlated with phakic eye presence (*p* < 0.05).

The occurrence of retinal nonperfusion was related to diffuse (*p* < 0.001) and focal (*p* < 0.001) macular edema presence. Diffuse macular edema was four times more frequent in patients with retinal nonperfusion (OR = 4.7, 95% CI: 2.0-11.0), while focal macular edema showed four times higher occurrence in patients without retinal nonperfusion (OR = 4.7, 95% CI: 2.0-11.0).

Similarly, our analyses showed that retinal nonperfusion was correlated with vascular leakage (*p* < 0.001 for all three examined zones: far-periphery, midperiphery, and posterior zones). We found that retinal nonperfusion increases the chance for a vascular leakage by about twenty-six times (OR = 26.4, 95% CI: 7.2-97.3) in the far-periphery zone of the retina, almost sixty times (OR = 58.8, 95% CI: 12.5-275.4) compared to that in the midperiphery zone and about five times compared to that in the posterior retina (OR = 5.2, 95% CI: 2.2-12.4).

Retinal nonperfusion also correlated with the severity of nonproliferative diabetic retinopathy (NPDR) (*p* < 0.001). Approximately half (51%) of each group of patients, with and without retinal nonperfusion, was diagnosed with moderate NPDR. Mild NPDR was observed more frequently in diabetic patients without observed retinal nonperfusion (49% *vs.* 4% for patients with retinal nonperfusion), whereas severe NPDR was observed in patients with diagnosed retinal nonperfusion (45% *vs.* 0% for patients without observed nonperfusion).

The occurrence of retinal nonperfusion was not related to the ongoing insulin (*p* = 0.414) or anticoagulant treatment (*p* = 0.073), the presence of hypertension (*p* = 0.134) or coronary heart disease (*p* = 0.789), and the elevated cholesterol levels (*p* = 0.154). However, it was negatively correlated with renal failure (*p* < 0.05). No patient without observed nonperfusion reported renal failure, and 14% of patients with retinal nonperfusion reported experiencing renal failure at any stage of diabetes.

All patients included in the study were suffering from type 2 diabetes mellitus. Our analyses showed that the patients without retinal nonperfusion differed in age from patients with retinal nonperfusion (*p* < 0.05), and they were on average 2.9 years older (95% CI: 0.1-5.8).

Body mass index (BMI) of the patients without retinal nonperfusion was the same as BMI of patients with retinal nonperfusion (*p* = 0.736), but these two groups of patients differed in the duration of type 2 diabetes mellitus (T2DM) (*p* < 0.05) and in the glycated hemoglobin (HbAc1) level (*p* < 0.001) (Table 3). Simultaneously, HbA1c level in patients with retinal nonperfusion was higher by about 0.5 mg/dl (95% CI: 0.3-0.7) as compared to patients without retinal nonperfusion.

### 3.2. Effect of Long-Term Bevacizumab Treatment on the Retinal Nonperfusion Areas

Forty-nine of the patients included in the study were diagnosed with retinal nonperfusion.

Our results show that intravitreal treatment with bevacizumab reduced the area of retinal nonperfusion in the patients with diabetic macular edema (DME). The area of the retinal nonperfusion was significantly smaller after 9 bevacizumab injections in the far-periphery (*p* < 0.001), midperiphery (*p* < 0.001), and posterior (*p* < 0.001) zones of the retina and in the total retinal area (*p* < 0.001) (Table 4).

Total area of retinal nonperfusion negatively correlated with BCVA (best-corrected visual acuity) before (high correlation, *ρ* = −0.544) and after (high correlation, *ρ* = −0.500) bevacizumab treatment (Table 5). Reversely, total area of retinal nonperfusion positively correlated with macular central subfield thickness (CST). The correlation was high before (*ρ* = 0.672) and average after (*ρ* = 0.493) bevacizumab treatment. We found that a larger total area of retinal nonperfusion corresponded to a greater total microaneurysm (MA) count (average correlation, *ρ* = 0.380) and a greater MA count in the posterior retinal zone (average correlation, *ρ* = 0.491) before the treatment and after it (average correlation, *ρ* = 0.482 for total MA count; high correlation, *ρ* = 0.588 for MA count in the posterior retinal zone). Also, a greater total area of retinal nonperfusion corresponded to a greater total leakage area (high correlation, *ρ* = 0.522), far-periphery leakage area (average correlation, *ρ* = 0.490), and midperiphery leakage area (average correlation, *ρ* = 0.450) before bevacizumab treatment and after it (average correlation for all three parameters: *ρ* = 0.468, *ρ* = 0.365, and *ρ* = 0.393, respectively). There was no statistically significant correlation between the total area of retinal nonperfusion and the leakage area in the posterior zone of the retina (*p* = 0.5255).

In order to present the relationships between the analyzed variables, we calculated the relative parameters (Table 5) according to equation (3) presented in Section 2.6. We found that the decrease in the total area of retinal nonperfusion was related to the decrease in the macular CST (average correlation, *ρ* = 0.340), the decrease in total MA count (high correlation, *ρ* = 0.554), the decrease in the MA count in the posterior retinal zone (average correlation, *ρ* = 0.479), the decrease in the total leakage area (high correlation, *ρ* = 0.607), and the decrease in the leakage area in the posterior retinal zone (high correlation, *ρ* = 0.541). We also found that the decrease in the far-periphery nonperfusion area was accompanied by the decrease in the total MA count (average correlation, *ρ* = 0.360).

### 3.3. Effect of Long-Term Bevacizumab Treatment on Best-Corrected Visual Acuity (BCVA)

We found that best-corrected visual acuity (BCVA) improved after bevacizumab treatment regardless of the retinal nonperfusion presence (*p* < 0.001) (Table 6). However, the analyses showed no effect of retinal nonperfusion (*p* = 0.165) and no interaction between the study time and retinal nonperfusion (*p* = 0.935) (Table 6). Analyzing each group of patients separately before and after bevacizumab treatment, we found no statistically significant differences in BCVA improvement (*p* = 0.239 and *p* = 0.163, respectively).

The analyses showed that the ETDRS score significantly depended on the therapy in both groups of patients with (*χ*^2^ = 41.79; *p* < 0.001) and without retinal nonperfusion (*χ*^2^ = 35.93; *p* < 0.001). The number of people showing the same change in BCVA test score was similar in both groups of patients (*χ*^2^ = 1.21; *p* = 0.751) (Table 7).

### 3.4. Effect of Bevacizumab Treatment on Macular Central Subfield Thickness (CST)

The analyses of macular central subfield thickness (CST) showed that bevacizumab treatment reduced macular edema (*p* < 0.001), and this effect did not depend on retinal nonperfusion presence (*p* = 0.859) (Table 6). CST in patients with and without retinal nonperfusion was the same before (*p* = 0.852) and after (*p* = 0.936) bevacizumab treatment, when analyzed separately for each group (Table 6).

In the group of patients with diagnosed retinal nonperfusion, the administration of earlier doses of bevacizumab had a more substantial effect on macular CST reduction. We found significant differences in macular CST between patients with and without retinal nonperfusion. These differences were recorded for measurements taken at subsequent, early (from the 2nd to the 4th) appointments when following injections of the bevacizumab were administered. We could observe the positive results of the bevacizumab treatment immediately after the first injection (Table 8).

### 3.5. Effect of Bevacizumab Treatment on Retinal Microaneurysm (MA) Count

Analysis of total MA count and MA count in the posterior zone of the retina showed statistically significant differences after the treatment (for both variables, *p* < 0.001), and retinal nonperfusion occurrence (for both variables, *p* < 0.001) (Table 6). We also found interactions between the treatment and retinal nonperfusion occurrence (*p* < 0.001 for total MA count and *p* < 0.05 for MA count in the posterior zone of the retina). Also, total MA count and MA count in the posterior zone of the retina recorded for patients with retinal nonperfusion before bevacizumab treatment were higher than in patients without retinal nonperfusion at the same time (*p* < 0.001). After the therapy, values for both variables decreased in both groups. In the case of total MA count, the values differed between both analyzed groups (*p* < 0.001), while for MA count in the posterior zone of the retina, the values were similar in both analyzed groups (*p* = 0.077) (Table 6). We conclude that bevacizumab therapy was equally effective for diabetic patients with and without retinal nonperfusion, when assessed with MA count in the posterior zone of the retina.

### 3.6. Effect of Bevacizumab Treatment on Leakage Area

Our analyses showed statistically significant differences in total leakage area before and after bevacizumab treatment (*p* < 0.001) (Table 6). Differences resulting from retinal nonperfusion occurrence (*p* < 0.001) were found regardless of the treatment, and no interaction between the treatment and nonperfusion occurrence was recorded (*p* = 0.086). Significantly higher values of total leakage area were noted in the group of patients with retinal nonperfusion before bevacizumab treatment (*p* < 0.001), and this tendency was noted when comparing the two groups after the treatment (*p* < 0.001).

Analyses for the far-periphery zone showed a significant reduction in leakage area in both groups of patients (*p* < 0.05 for patients with nonperfusion; *p* < 0.001 for patients without nonperfusion) (Table 6). Patients with retinal nonperfusion had a larger leakage area in the far-periphery zone before bevacizumab treatment when compared to the group of patients without retinal nonperfusion (*p* < 0.001), while after the treatment, the leakage areas did not differ statistically (*p* = 0.83, Table 6).

The leakage area in the midperiphery and posterior zones of the retina before and after bevacizumab treatment differed significantly between the groups (for both variables and groups, *p* < 0.001). The treatment reduced the leakage area in each of the analyzed zones in both groups of patients. Also, the leakage areas in the midperiphery (*p* < 0.001) and posterior zones of the retina (*p* < 0.001) were always (before and after the treatment) larger in the group of patients diagnosed with retinal nonperfusion (for the midperiphery zone before and after treatment, *p* < 0.001; for the posterior zone before treatment, *p* < 0.001; and after treatment, *p* < 0.05) (Table 6).

### 3.7. Effect of Bevacizumab Treatment in Particular Areas of the Retina in Patients with Retinal Nonperfusion

We found a high positive correlation between the treatment effect assessed with the BCVA test and the total area of nonperfusion (*p* < 0.001) (Table 9), and average positive correlations with the NPA in the far-periphery and the midperiphery zones of the retina (*p* < 0.01 for both zones). Simultaneously, we noted statistically significant highly negative correlation between the treatment effect assessed with macular central subfield thickness (CST) and the total area of retinal nonperfusion (*p* < 0.001), and average negative correlations with the NPA in the far-periphery and midperiphery retinal zones (*p* < 0.01 for both zones). The treatment effect assessed with relative BCVA and relative CST did not correlate with the retinal nonperfusion area in the posterior zone of the retina (*p* = 0.760 and *p* = 0.740, respectively). The effect of bevacizumab treatment did not depend on the area of nonperfusion in the posterior zone of the retina, and it was positive regardless of the size of the nonperfusion area.

## 4. Discussion

In this study, we analyzed the relation between the size of retinal nonperfusion areas (NPA) and the effectiveness of 12-month intravitreal bevacizumab (IVB) therapy in patients with treatment-naïve diabetic macular edema (DME). The effectiveness of the treatment was assessed using ultra-wide-field fluorescein angiography (UWFFA) and swept-source optical coherence tomography (SS-OCT). The results showed that (i) retinal NPA decreased in the far-periphery, midperiphery, and posterior zones of the retina during bevacizumab treatment in patients with DME; (ii) there was no progression of diabetic retinopathy in patients with DME after the bevacizumab treatment; (iii) the size of retinal NPA was associated with diabetic retinopathy severity, but bevacizumab treatment stabilized or reversed it; (iv) patients with DME responded well to bevacizumab treatment regardless of the presence of retinal nonperfusion—retinal nonperfusion did not affect the efficacy of bevacizumab treatment aimed at reducing DME; (v) peripheral retinal nonperfusion did not affect the pattern of visual acuity, the severity of DME, and the efficacy of bevacizumab therapy; (vi) bevacizumab treatment was equally effective in treating microaneurysms in the posterior zone of the retina in patients with and without retinal nonperfusion; (vii) patients with retinal nonperfusion were more frequently diagnosed with diffuse macular edema, while patients without retinal nonperfusion were diagnosed with focal macular edema; (viii) retinal nonperfusion was associated with vascular leakage; and (ix) patients with retinal nonperfusion had faster progression of diabetic retinopathy compared to patients without retinal nonperfusion and higher HbA1c (glycated hemoglobin) levels.

The UWFFA effectively detects the peripheral retinal nonperfusion that correlates with increased risk of DME in patients with diabetic retinopathy (DR) [23, 24]. In this study, we used UWFFA with a 200° field view in a single image. We demonstrated that intravitreal bevacizumab treatment strongly reduced the NPA in the far-periphery, midperiphery, and posterior zones of the retina in the analyzed groups of patients with DME, when comparing the results before and after the treatment. The most effective changes in NPA occurred in the midperiphery zone of the retina. Xue et al. also reported a positive response to 4-month anti-VEGF treatment with ranibizumab in patients with DME with significant peripheral retinal nonperfusion. They observed that the reduction of absolute and relative central retinal thickness was associated with the number of microaneurysms, peripheral nonperfusion, and neovascularization [25]. Wykoff et al. showed that aflibercept treatment not only slowed the worsening of retinal perfusion but also improved it in some cases by decreasing areas of retinal nonperfusion [26]. Since Yoo et al. observed that macular edema recurred in 65.1% of the eyes after initial bevacizumab (IVB) injection [27], we hope that our approach (9 injections over 12 months) will ensure long-lasting effects in the studied groups of patients. We observed that macular CST significantly decreased after three initial doses of bevacizumab, more in patients with retinal NPA than in patients without NPA. The next doses maintained the reductive effect of bevacizumab, but we would need to investigate further if the effect lasts after this 12-month treatment. The odds are high as Cai et al. proved the therapeutic efficiency of anti-VEGF agents injected 9-10 times during the first year and 5-6 times during the second year of DME treatment [18].

UWFFA showed that peripheral retinal nonperfusion did not affect the best-corrected visual acuity (BCVA), the severity of DME, and the efficacy of bevacizumab treatment. The total NPA was highly positively correlated with macular CST (central subfield thickness) and highly negatively correlated with BCVA in the group of patients with retinal nonperfusion. BCVA after the treatment was better both in patients without retinal nonperfusion and with retinal nonperfusion, but the effects of the therapy did not differ significantly between the analyzed groups. Bevacizumab treatment applied in our study had a positive effect on visual acuity. This does not correspond with other studies reporting that macular NPA may have hindered the visual acuity in patients with DME 3 months after intravitreal bevacizumab injections [28]. The difference may result from a different time at which the visual acuity was evaluated. In our study, we evaluated it one month after the last bevacizumab injection. Nevertheless, other reports confirm that a thoroughly selected approach offers the opportunity to individualize management while minimizing the burden of the treatment [29–31].

Our study showed that bevacizumab treatment reduced macular CST, but the effect was independent of the NPA presence. Intergroup comparison between patients with retinal nonperfusion and those without it showed no difference in macular CST between these groups before and after bevacizumab treatment. Mushtaq et al. also demonstrated a significant improvement in central retinal thickness after bevacizumab treatment. They reported that the greatest improvement was observed in the group of patients with central retinal thickness > 400 *μ*m [32]. Other authors, like Yoo et al. [27], studying patients with branch retinal vein occlusion with nonperfusion larger than a 0.5 mm ETDRS (Early Treatment Diabetic Retinopathy Study) circle zone or with an initial central retinal thickness > 570 *μ*m, noticed that these patients should be closely monitored after the treatment. Their results showed that macular edema recurred within six months after a single bevacizumab injection in most patients [27]. This agrees with our observation that a more frequent injection regimen is a key factor for the positive outcome of the treatment. The other studies showed that bevacizumab treatment resulted in a better visual outcome regardless of initial macular edema and initial central macular thickness but only at 6 weeks [33]. In our study, the average macular CST in both analyzed groups before bevacizumab treatment was >400 *μ*m; after bevacizumab treatment, it was <300 *μ*m. This change was accompanied by an improvement in the BCVA score by about 10 ETDRS letters also in both analyzed groups. More importantly, the effects of bevacizumab treatment on DME reduction did not depend on the retinal nonperfusion presence. First doses of bevacizumab had a more substantial effect on macular CST reduction in patients with retinal nonperfusion: macular CST was lower in patients with retinal nonperfusion than in patients without nonperfusion after injections I-III, and this pattern was not observed after injections IV-IX. The randomized clinical trials examining severe disease reported a significant improvement in BCVA and reduction of central macular thickness after three intravitreal injections with bevacizumab. However, the effect decreased gradually as both measured variables returned to near-baseline values 3 months after the last intravitreal injection [34]. Several reports indicate that bevacizumab injections may have a beneficial effect on macular thickness and visual acuity in diffuse DME [35]. According to authors, bevacizumab monotherapy improves visual function, measured by fluorescein angiography and optical coherence tomography, and stabilizes diabetic macular edema [35]. On the contrary, Jeon and Lee reported that continuous intravitreal bevacizumab monotherapy had no beneficial effect on visual acuity and the number of hard exudates 6 months after the patients with DME with subfoveal and perifoveal hard exudates completed the treatment. In that study, anti-VEGF treatment did not facilitate lipid or proteinaceous material resorption, which would be the expected effect of treatment, as hard exudates are related to photoreceptor and neuronal degeneration in the outer plexiform layer and their presence increases the risk of visual impairment [36].

DME occurs when the barrier between the blood and the retina is damaged and vascular fluid and proteins leak and accumulate in the macula. Studies show that DME is related to microaneurysm (MA) and retinal leakage presence. It is known that both changes present similar spatial distribution in the retina. This indicates that leakage emerges from strongly hyperpermeable microaneurysms in the course of diabetic retinopathy [37]. Our results showed that the size of NPA was related to the total microaneurysm (MA) count and MA count in the posterior zone of the retina. The total MA count in both groups of patients was related to the treatment duration, nonperfusion presence, and interaction between those two factors. We observed a significant reduction in the total MA count before and after therapy. The effect of bevacizumab treatment was significantly stronger in patients with retinal nonperfusion than in patients without it. However, when it comes to MA count in the posterior zone of the retina, we can conclude that the bevacizumab treatment was equally effective for patients with and without retinal nonperfusion.

Additionally, we noted that an increase in the total nonperfusion area (NPA) had an impact on the increase in the total leakage, far-periphery leakage, and midperiphery leakage areas. We found that retinal nonperfusion was related to peripheral and posterior vascular leakage. Feng et al. reported that the nonproliferative diabetic retinopathy with leakage (expressed with leakage index) varied depending on the region of the retina and between them. They also noted that the leakage index decreased as the distance from the fovea increased. The highest leakage was observed in the posterior and midperiphery zones of the retina [38]. Silva et al. observed the greatest amount of nonperfusion in the midperiphery zone of the retina [4]. On the contrary, Kristinsson et al. [39] observed that nonproliferative retinopathy with leakage was more extensive in the posterior zone of the retina than in its peripheral zones. The posterior part of the retina has the highest vascular density which translates to higher overall metabolic activity. These features make this region of the retina more susceptible to leakage [39]. In our study, the leakage was higher in the group of patients with nonperfusion than in the group of patients without it. Bevacizumab treatment significantly reduced vascular leakage, which acknowledged the efficiency of anti-VEGF treatment in patients with DME. Niki et al. also reported that an early stage of diabetic retinopathy, which in this case is nonproliferative diabetic retinopathy without leakage, was more extensive in the midperiphery than in other regions of the retina [5]. This finding agreed with previous analyses of overall nonproliferative diabetic retinopathy. The analyses showed that the condition is usually first recognized in the midperiphery zone of the retina. The authors suggested that the midperipheral retina in diabetic eyes may be more prone to develop capillary closure than its posterior part [5, 40].

Our analyses of UWFFA images showed that diffuse macular edema was more frequent in patients with retinal nonperfusion, while focal macular edema was more frequent in patients without retinal nonperfusion. This explains the fact that both the leakage area and the MA count were higher in patients with retinal nonperfusion than in patients without it. The diffuse macular edema was responding well to bevacizumab treatment. Bevacizumab treatment reduced vascular leakage in patients with retinal nonperfusion; thus, it can be considered an effective treatment in this group of patients.

Our study showed that diabetic retinopathy did not progress in patients with diagnosed DME after 9 injections (12 months) of bevacizumab treatment. We can conclude that patients with retinal nonperfusion present faster progress of the disease when compared to patients without retinal nonperfusion. In our study, the retinal nonperfusion was related to the diabetic retinopathy severity, but bevacizumab treatment stabilized it or even reversed it. This agrees with other studies that reported the usefulness of bevacizumab intravitreal injection in DME reduction. The studies reported that consecutive bevacizumab monotherapy, or combined with intravitreal triamcinolone, resulted in anatomic and functional improvement of the retina, and the results were compared with macular focal or grid laser photocoagulation [33, 41]. Another semiquantitative study, in a small series of patients with DR, showed that unselective intravitreal bevacizumab treatment improved peripheral nonperfusion in the short term [42]. Cataract surgery combined with bevacizumab injections seems to be an effective treatment option in patients with coexisting diabetic retinopathy [38]. Bevacizumab treatment administered immediately after cataract surgery represents a safe and effective strategy: it prevents postoperative macular thickening or reduces macular edema, and it improves average visual acuity in diabetic patients [43].

In this study, we used UWFFA to evaluate the effectiveness of bevacizumab treatment in two groups of patients suffering from diabetic retinopathy and diabetic macular edema (DME): one group with retinal nonperfusion and another group without. The strict regimen of intravitreal injections was equally effective in both analyzed groups of patients, which is very important for proper ophthalmic management. Many reports compared therapeutic bevacizumab injections with retina laser therapy and injections combined or laser therapy alone. Peripheral retinal laser photocoagulation may be performed only if the nonperfusion areas are present. The procedure significantly reduces retinal nonperfusion but leaves the retina with many coagulated spots after laser application, which causes local destruction of the retina. In this study, the strict regimen of bevacizumab injections decreased DME and diabetic retinopathy severity in both groups of patients, one with retinal nonperfusion and the other without. Additionally, no macular grid laser photocoagulation was needed during this intensive course of treatment. However, it can be expected that some patients would need it in the future. UWFFA, as a modern diagnostic method, allowed us to give a better prognosis to patients treated with anti-VEGF with retinal nonperfusion. The main limitations of this study are connected to the number of patients qualified for the study and the lack of information about the long-term effects of the treatment.

## 5. Conclusions

The strict regimen of bevacizumab injections decreased diabetic macular edema (DME) and the severity of diabetic retinopathy in two groups of patients, one with retinal nonperfusion and the other without. The applied protocol of bevacizumab reduced the retinal nonperfusion areas. Patients without retinal nonperfusion showed no progression in diabetic retinopathy. UWFFA allowed us to determine a satisfactory prognosis for patients with retinal nonperfusion. For this group of patients, bevacizumab treatment was as effective as in patients without nonperfusion. Our study confirmed that patients with DME could be successfully treated with bevacizumab independently from nonperfusion status.

## Figures and Tables

**Table 1:** The inclusion and exclusion criteria for patients with diabetic macular edema participating in the study evaluating the effectiveness of intravitreal bevacizumab treatment.

Inclusion criteria	Exclusion criteria
≥18 years old	Previous intravitreal anti-VEGF or steroid therapy

Diabetes mellitus type 1 or 2	Retinal photocoagulation

DME with CST ≥ 250 *μ*m	History of pars plana vitrectomy or cataract surgery with posterior capsule rupture

Nonproliferative diabetic retinopathy	Media opacity disabling to assess the fundus of the eye

BCVA of 24-78 ETDRS letters	Vitreoretinal traction in the macula
	Epiretinal membrane influencing BCVA
	Proliferative diabetic retinopathy
	Systemic treatment influencing the retina or DME
	Nonperfusion of the foveal area
	Any concurrent nondiabetic retinal disease

Abbreviation: CST = central subfield thickness.

**Table 2:** Comparison of diabetic retinopathy qualitative variables of patients with diabetic macular edema (DME) without (*N* = 49) or with (*N* = 49) diagnosed retinal nonperfusion qualified for intravitreal bevacizumab treatment. The results are presented as mean ± standard deviation using the chi-squared (*χ*^2^) test. Statistical significance was set at *p* < 0.05.

Variable	Variant	Patients without nonperfusion (*N* = 49)	Patients with nonperfusion (*N* = 49)	*χ* ^2^	*p*
Sex	Woman	29 (59%)	28 (57%)	0.04	0.838
	Man	20 (41%)	21 (43%)		

Eye	Right	21 (43%)	25 (51%)	0.66	0.418
	Left	28 (57%)	24 (49%)		

Lens status	Phakic	19 (39%)	31 (63%)	5.88	**<0.05**
	Pseudophakic	30 (61%)	18 (37%)		

Diabetic macular edema	Diffuse	16 (33%)	34 (69%)	13.23	**<0.001**
	Focal	33 (67%)	15 (31%)		

Vascular leakage (far-periphery zone)	Yes	3 (6%)	31 (63%)	35.31	**<0.001**
	No	46 (94%)	18 (37%)		

Vascular leakage (midperiphery zone)	Yes	2 (4%)	35 (71%)	47.28	**<0.001**
	No	47 (96%)	14 (29%)		

Vascular leakage (posterior zone)	Yes	17 (35%)	36 (73%)	14.83	**<0.001**
	No	32 (65%)	13 (27%)		

Nonproliferative diabetic retinopathy	Mild	24 (49%)	2 (4%)	40.62	<0.001
	Moderate	25 (51%)	25 (51%)		
	Severe	0 (0%)	22 (45%)		

Insulin treatment	Yes	26 (53%)	30 (61%)	0.67	0.414
	No	23 (47%)	19 939%)		

Hypertension	Yes	36 (73%)	29 (59%)	2.24	0.134
	No	13 (27%)	20 (41%)		

Hypercholesterolemia	Yes	31 (63%)	24 (49%)	2.03	0.154
	No	18 (37%)	25 (51%)		

Coronary heart disease	Yes	8 (16%)	9 (18%)	0.07	0.789
	No	41 (84%)	40 (82%)		

Renal failure	Yes	0 (0%)	7 (14%)	—	**<0.05**
	No	49 (100%)	42 (86%)		

Anticoagulant treatment	Yes	7 (14%)	2 (4%)	3.22	0.073
	No	42 (86%)	47 (96%)		

Abbreviations: DME = diabetic macular edema.

**Table 3:** Comparison of diabetic retinopathy quantitative variables of patients with diabetic macular edema (DME) without (*N* = 49) or with (*N* = 49) diagnosed retinal nonperfusion qualified for long-term intravitreal bevacizumab treatment. *t*-test for two independent samples. The results are presented as mean ± standard deviation. Statistical significance was set at *p* < 0.05.

Variable	Patients without nonperfusion (*N* = 49)	Patients with nonperfusion (*N* = 49)	*t*	*p*
Age (years)	70.0 ± 5.5	67.1 ± 8.4	2.02	**<0.05**
BMI (kg/m^2^)	26.4 ± 1.8	26.3 ± 1.9	0.34	0.736
HbA1c serum concentration (mg/dl)	6.6 ± 0.4	7.0 ± 0.5	4.62	**<0.001**

Abbreviations: T2DM = type 2 diabetes mellitus; BMI = body mass index; HbA1c = glycated hemoglobin.

**Table 4:** Comparison of retinal nonperfusion area according to retinal zone before and after bevacizumab intravitreal treatment in patients with diabetic macular edema (DME) (*N* = 49) using the Wilcoxon signed-rank test. The results are presented as median (lower-upper quartile). Statistical significance was set at *p* < 0.05.

Nonperfusion area according to retinal zone (mm^2^)	Before bevacizumab therapy	After bevacizumab therapy	Δ = before − after	*Z*	*p*
Total retinal area	29 (14-36)	12 (4-18)	12 (5-24)	5.86	**<0.001**
Far periphery	10 (0-23)	2 (0-10)	2 (0-11)	4.37	**<0.001**
Midperiphery	6 (3-10)	1 (0-4)	4 (1-8)	5.11	**<0.001**
Posterior	4 (1-11)	1 (0-5)	2 (0-5)	4.46	**<0.001**

**Table 5:** Spearman's rank correlation (Spearman's *ρ*) between examined parameters in the total retinal area and far-periphery retinal area before and after bevacizumab intravitreal treatment of patients with diabetic macular edema (DME) and with diagnosed retinal nonperfusion (*N* = 49). Statistical significance was set at *p* < 0.05.

Relative retinal nonperfusion area	Examined relative parameter		
		*ρ*	*p*
(Total retinal NPA)_relative_	BCVA_relative_	-0.071	0.631
	CST_relative_	0.340	**<0.05**
	(Total MA count)_relative_	0.554	**<0.001**
	(MA count in posterior)_relative_	0.479	**<0.001**
	(Total leakage area)_relative_	0.607	**<0.001**
	(Leakage area in far periphery)_relative_	0.143	0.384
	(Leakage area in midperiphery)_relative_	0.219	0.159
	(Leakage area posterior)_relative_	0.541	**<0.001**

(Far − periphery NPA)_relative_	BCVA_relative_	0.037	0.835
	CST_relative_	0.162	0.359
	(Total MA count)_relative_	0.360	**<0.05**
	(MA count in posterior)_relative_	0.337	0.051
	(Total leakage area)_relative_	0.318	0.067
	(Leakage area in far periphery)_relative_	0.256	0.181
	(Leakage area in midperiphery)_relative_	0.106	0.583
	(Leakage area posterior)_relative_	0.200	0.256

*ρ*: Spearman's rank correlation coefficient; BCVA: best-corrected visual acuity; CST: central subfield thickness; MA: microaneurysm; NPA: nonperfusion area.

**Table 6:** Comparison of examined parameters of patients with diabetic macular edema (DME) without (*N* = 49) or with (*N* = 49) diagnosed retinal nonperfusion before and after intravitreal bevacizumab treatment using two-way analysis of variance. The results are presented as mean ± standard deviation or median (lower-upper quartile). Statistical significance was set at *p* < 0.05.

Examined parameters	Study group	Before bevacizumab therapy	After bevacizumab therapy	*p*_time_	*p*_nonperfusion_	*p*_interactions_
BCVA (ETDRS letters)	Patients *without* nonperfusion	68.0 ± 7.3	76.8 ± 5.6	**<0.001**	0.165	0.935
	Patients *with* nonperfusion	66.1 ± 8.7	74.7 ± 8.4			
	*p*_without nonperfusion vs. with nonperfusion_	0.239	0.163			

CST (*μ*m)	Patients *without* nonperfusion	419.3 ± 86.1	287.3 ± 49.7	**<0.001**	0.859	0.888
	Patients *with* nonperfusion	422.7 ± 94.8	288.2 ± 58.3			
	*p*_without nonperfusion vs. with nonperfusion_	0.852	0.936			

Total MA count (*N*)	Patients *without* nonperfusion	115.4 ± 35.5	71.1 ± 32.1	**<0.001**	**<0.001**	**<0.001**
	Patients *with* nonperfusion	188.2 ± 60.7	109.9 ± 50.7			
	*p*_without nonperfusion vs. with nonperfusion_	**<0.001**	**<0.001**			

MA count (*N*)—posterior zone	Patients *without* nonperfusion	84.7 ± 31.1	48.1 ± 27.9	**<0.001**	**<0.001**	**<0.05**
	Patients *with* nonperfusion	117.1 ± 49.2	59.8 ± 36.0			
	*p*_without nonperfusion vs. with nonperfusion_	**<0.001**	0.077			

Total leakage area (mm^2^)^∗^	Patients *without* nonperfusion	18.0 (12.0-24.0)	4.0 (2.0-6.0)	<0.001^∗^	**<0.001**	0.086
	Patients *with* nonperfusion	60.0 (32.0-107.0)	11.0 (4.0-16.0)			
	*p*_without nonperfusion vs. with nonperfusion_	**<0.001**	**<0.001**			

Leakage area in far-periphery zone (mm^2^)	Patients *without* nonperfusion	0.0 (0.0-0.0)	0.0 (0.0-0.0)	**<0.05**	—	—
	Patients *with* nonperfusion	8.0 (2.0-23.0)	0.0 (0.0-1.0)	**<0.001**		
	*p*_without nonperfusion vs. with nonperfusion_	**<0.001**	0.083			

Leakage area in midperiphery zone (mm^2^)	Patients *without* nonperfusion	0.0 (0.0-3.0)	0.0 (0.0-0.0)	**<0.001**		
	Patients *with* nonperfusion	11.0 (4.0-25.0)	2.0 (0.0-4.0)	**<0.001**		
	*p*_without nonperfusion vs. with nonperfusion_	**<0.001**	**<0.001**			

Leakage area in posterior zone (mm^2^)^∗∗^	Patients *without* nonperfusion	16.0 (9.0-21.0)	4.0 (2.0-6.0)	**<0.001**		
	Patients *with* nonperfusion	24.0 (16.0-69.0)	6.0 (3.0-12.0)	**<0.001**		
	*p*_without nonperfusion vs. with nonperfusion_	**<0.001**	**<0.05**			

^∗^Two-way ANOVA after log transformation of variables. ^∗∗^The Friedman test. BCVA: best-corrected visual acuity; CST: central subfield thickness; ETDRS: Early Treatment Diabetic Retinopathy Study; MA: microaneurysm.

**Table 7:** Change in best-corrected visual acuity (BCVA) of patients with diabetic macular edema (DME) without (*N* = 49) or with (*N* = 49) diagnosed retinal nonperfusion before and after bevacizumab treatment. The results are presented as the number of patients showing the change.

Change in BCVA	Patients without nonperfusion	Patients with nonperfusion
Deterioration	0	1
No improvement	11	9
Improvement by 10 ETDRS letters (1 aggregated unit)	33	34
Improvement by 20 ETDRS letters (2 aggregated units)	5	5

Abbreviations: BCVA = best-corrected visual acuity; ETDRS = Early Treatment Diabetic Retinopathy Study.

**Table 8:** Central subfield thickness (CST) at the fovea of the retina in patients with diabetic macular edema (DME) without (*N* = 49) or with (*N* = 49) retinal nonperfusion during subsequent intravitreal bevacizumab (0.5 mg/0.05 ml) injections. The results are presented as mean ± standard deviation. Statistical significance was set at *p* < 0.05.

Time of therapy (months)	Bevacizumab injection no	CST (*μ*m) in patients without nonperfusion (*N* = 49)	CST (*μ*m) in patients with nonperfusion (*N* = 49)	*p*_without nonperfusion vs. with nonperfusion_
0	I	419.3 ± 86.1	422.7 ± 94.8	0.852
1	II	376.8 ± 79.7	344.8 ± 78.4	**<0.05**
2	III	358.4 ± 71.8	316.3 ± 74.5	**<0.01**
3	IV	341.0 ± 75.5	307.5 ± 77.7	**<0.05**
4	V	316.8 ± 56.9	297.8 ± 62.7	0.120
6	VI	300.5 ± 62.0	299.3 ± 75.3	0.930
8	VII	293.0 ± 55.4	298.0 ± 71.4	0.704
10	VIII	293.0 ± 55.4	292.7 ± 61.5	0.871
12	IX	287.3 ± 49.7	288.2 ± 58.3	0.936

Abbreviation: CST = central subfield thickness.

**Table 9:** Spearman's rank correlation (Spearman's *ρ*) between relative best-corrected visual acuity (BCVA_relative_), relative central subfield thickness (CST_relative_), and zone of the retina in patients with diabetic macular edema (DME) with (*N* = 49) diagnosed retinal nonperfusion qualified for intravitreal bevacizumab treatment. Statistical significance was set at *p* < 0.05.

	Zone of retinal nonperfusion before therapy	*ρ*	*p*
BCVA_relative_ (%)	Total area of the retina	0.589	**<0.001**
	Far periphery	0.402	**<0.01**
	Midperiphery	0.441	**<0.01**
	Posterior	0.045	0.760

CST_relative_ (%)	Total area of the retina	-0.614	**<0.001**
	Far periphery	-0.432	**<0.01**
	Midperiphery	-0.427	**<0.01**
	Posterior	-0.048	0.740

*ρ*: Spearman's rank correlation coefficient.

## Data Availability

The numerical data is available after contact with the corresponding author.
